# Highly accurate diagnosis of pancreatic cancer by integrative modeling using gut microbiome and exposome data

**DOI:** 10.1016/j.isci.2024.109294

**Published:** 2024-02-21

**Authors:** Yuli Zhang, Haohong Zhang, Bingqiang Liu, Kang Ning

**Affiliations:** 1 School of Mathematics, Shandong University, Jinan 250200, Shandong, China; 2 Key Laboratory of Molecular Biophysics of the Ministry of Education, Hubei Key Laboratory of Bioinformatics and Molecular-imaging, Center of AI Biology, Department of Bioinformatics and Systems Biology, College of Life Science and Technology, Huazhong University of Science and Technology, Wuhan 430074, Hubei, China

**Keywords:** Environment, Microbiome, Cancer, Machine learning

## Abstract

The noninvasive detection of pancreatic ductal adenocarcinoma (PDAC) remains an immense challenge. In this study, we proposed a robust, accurate, and noninvasive classifier, namely Multi-Omics Co-training Graph Convolutional Networks (MOCO-GCN). It achieved high accuracy (0.9 ± 0.06), F1 score (0.9± 0.07), and AUROC (0.89± 0.08), surpassing contemporary approaches. The performance of model was validated on an external cohort of German PDAC patients. Additionally, we discovered that the exposome may impact PDAC development through its complex interplay with gut microbiome by mediation analysis. For example, *Fusobacterium hwasookii nucleatum*, known for its ability to induce inflammatory responses, may serve as a mediator for the impact of rheumatoid arthritis on PDAC. Overall, our study sheds light on how exposome and microbiome in concert could contribute to PDAC development, and enable PDAC diagnosis with high fidelity and interpretability.

## Highlights

• MOCO-GCN achieved high-precision and noninvasive diagnosis of PDAC
• Refining the intricate interplay between the exposome and microbiome in PDAC insights
• MOCO-GCN identified potential microbiome and exposome biomarkers for PDAC

## Graphical abstract

**Figure:**
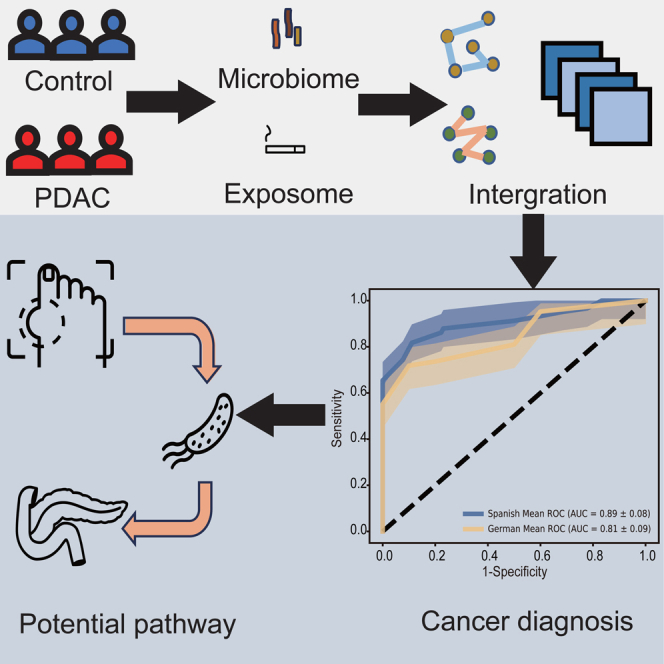

## Introduction

Pancreatic ductal adenocarcinoma (PDAC) is the fourth leading cause of death.^1^^,^^2^ It ranks firmly last among all cancer in terms of prognosis and only about 4% of patients would live five years after diagnosis as it often presents at an advanced stage.^3^^,^^4^ Recent studies have explored the PDAC biomarkers in tumor,^5^^,^^6^ blood,^7^ pancreatic tissue,^8^ urine,^9^ and serum.^10^ Currently, the only FDA-approved biomarker for pancreatic cancer is carbohydrate antigen (CA) 19-9; however, its specificity is limited by a high false positive rate, as its concentration may increase in benign diseases like gallstone and bile duct obstruction.^11^^,^^12^ Consequently, a noninvasive, robust, and accurate screening and diagnostic tool for PDAC is still urgently needed.

Numerous studies have explored links between PDAC and the oral^13^^,^^14^^,^^15^ or fecal microbiome.^16^^,^^17^ Nagata et al. conducted a multinational study and accurately predicted PDAC using 30 gut and 18 oral microbial species, achieving high area under the receiver operating characteristic (AUROCs) of 0.78–0.82.^16^ Kartal et al. proposed a fecal metagenomic classifiers based on 27 gut microbial species that could identify PDAC with high accuracy (0.84 AUROC) and validated the classifier in an independent German cohort (0.83 AUCROC) and confirmed the specificity in 25 publicly available studies.^17^ Additionally, Half et al.^18^ and Ren et al.^19^ employed the random forest to predict PDAC with high accuracy of 0.825 and 0.842 AUROC. These studies have shown that microbiota-based screening for the detection of PDAC is feasible.

The exposome is the comprehensive collection of all exposures, including smoking, alcohol, diet, exercise, other lifestyle factors, medication, host diseases, and more.^20^ Risk factors associated with the development of PDAC include alcohol,^21^ advancing age,^22^^,^^23^ smoking,^24^ family history,^25^^,^^26^ diabetes,^27^^,^^28^ obesity,^29^ etc. Changes in the gut microbiome can both affect and mediate the effects of exposome on the risk of PDAC. For example, dysbiosis of the microbiota has been linked to an increased incidence of obesity,^30^ with signaling pathways leading to NF-kB activation, contributing to inflammatory agents.^31^ Conversely, exposures that affect pancreatic tumor evolution could also affect the gut microbiome. Phillip et al. suggested that long-term alcohol consumption could induce the dysbiosis of Firmicutes and Bacteroidetes, which are enriched or depleted in PDAC.^32^ Meanwhile, physical activity may protect against PDAC by increasing the abundance of SCFA-producing bacteria.^33^ Therefore, the microbiome and exposome could in concert influence the metabolic and immune pathways of PDAC.

Pancreatic cancer used to be considered a localized disease because of its occurrence in pancreas tissue, an organ in the abdomen that lies behind the lower part of stomach.^34^ However, the current understanding of PDAC is that it is not solely a tumor microenvironment issue but also a systemic and environmental disease that involves both the microbiome and exposome. The possible pathways of microbiome and exposome interactions that influence pancreatic carcinogenesis are shown in Figure S1A. Treatment efficiency and adverse effects can differ vastly between individuals due to differences in age, sex, and environmental factors. The aim of precision medicine is thus to design the most appropriate intervention based on the biological information of each individual. Most existing efforts focus on exploring the role of microbiome in PDAC, but this approach oversimplifies the complexity of biological systems. Exposome also affects complex molecular pathways where different biological layers interact with each other. Consequently, although the development of PDAC should be considered as a systemic and environmental disease that includes factors from both exposome and microbiome, studies of pancreatic cancer have rarely been conducted to consider the combination of exposome and microbiome for its diagnosis.

In this study, we presented a robust, accurate, and noninvasive classifier based on the combination of exposome and microbiome data for PDAC diagnosis. Our cohort consisted of patients with detailed host variables, including subject characteristics, lifestyle factors, oral health status, medication use, and other host disease status. We found significant differences in gut microbiome profiles between PDAC and controls, as determined by microbiome-associated statistical and machine-learning analyses. Through mediation analyses, we revealed putative causal relationships between PDAC, the gut microbiome and exposome. For example, *Fusobacterium hwasookii nucleatum* can mediate the impact of rheumatoid arthritis on PDAC. Next, we applied the MOCO-GCN, which enables omics-specific and cross-omics association learning, for effective PDAC classification. Our classifier achieved excellent discrimination performance, with a combination of 125 microbial species and 23 exposures resulting in with 0.9 ± 0.06 ACC, 0.9± 0.07 F1, 0.89± 0.08 AUROC, 0.86± 0.13 Sn, 0.93± 0.10 Sp, and 0.80± 0.11 MCC. MOCO-GCN also achieved high prediction performance on an external cohort (AUROC = 0.81± 0.09). Our findings about changes in microbiome abundance (enriched or depleted) in PDAC and exposures are consistent with previous studies, including the increase of *Fusobacterium hwasookii nucleatum* and depletion of *Faecalibacterium prausnitzii.* MOCO-GCN outperformed traditional machine learning and other multi-omics classification methods, and our results were also better than those from previous studies that predicted PDAC with microbiome alone. These results highlight the importance of considering both exposome and microbiome data for PDAC diagnosis. Overall, our study provides valuable insights into the complex interplay between the exposome and microbiome and their contribution to the development of PDAC. The complete workflow of this study is illustrated in Figure S1B.

## Results

### Data collection and preparation

To predict PDAC by combining exposome and microbiome data, we collected 107 metagenomic samples from Spanish and 76 samples from German based on fecal microbial species.^17^ Missing values in the German metadata were imputed using the missForest algorithm, a random forest-based method for missing data imputation.^35^ The imputation process involved the construction of 100 trees to accurately estimate and fill in the missing values. Of these, every metadata was able to be defined as a binary variable with a positive and negative class, for example, alcohol was considered positive for drinkers and negative for non-drinkers. Full definitions and descriptions are shown in Table S3. This study involved PDAC patients with an age distribution ranging from 38 to 93 years old. Among the 107 patients, 67 were females, and 40 were males. The smoking status included ex-smokers, smokers, and non-smokers. Alcohol consumption was categorized into drinkers and non-drinkers. The patients' other health conditions, such as high blood pressure, diabetes, gum recession, among others, were recorded, totaling 10 different health conditions. Additionally, information on medication use included the usage of seven drugs, including aspirin, antibiotics, and so on.

### Impact of exposome on microbial composition in pancreatic cancer patients

To identify the impact of exposome on microbial composition in pancreatic cancer, we used gut microbiome data from Spanish,^17^ which included 57 PDAC patients and 50 controls, along with detailed 24 host variables (Figure S1C). Each metadata was classified as binary, with positive and negative classes. Our dataset comprised 24 host metadata variables categorized into four groups: subject characteristics, lifestyle factors, oral health, medication use, and host disease. Most of these variables are known risk factors for pancreatic cancer. Subsequently, our aim was to determine whether there were disparities in the distribution of microbial composition among the participants based on variations in host variables.

**Figure 1:**
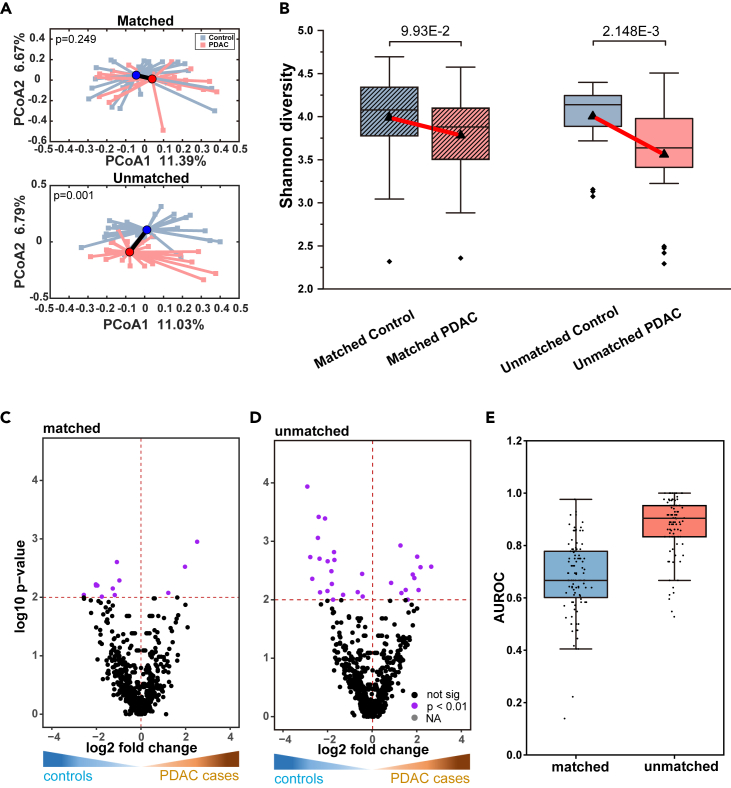
Variation of the matched and unmatched cohort in microbiota due to confounding variables between PDAC and controls (A) Principal coordinates analysis (PCoA) plot of PDAC and controls in the confounding-unmatched and matched cohort, with the PERMANOVA p value. The centroids for the PDAC and controls are depicted by an outlined circle. Colors denote groups, with blue for controls and red for PDAC patients. (B) Alpha diversity measurements comparing PDAC and controls in the unmatched and matched cohort. It was calculated as the Shannon index. Colors denote groups, with blue for controls and red for PDAC patients. Pairwise comparisons were performed using the Wilcoxon test. (C and D) The difference abundance analysis between PDAC and controls in the matched and unmatched cohort. It was implemented by the Wilcoxon test. Y-axis is log10 (p values), X axis is generalized fold change. Purple dots represent significantly differentially abundant in either group, while black dots show non-significant species. (E) Random forest AUROC values for PDAC and controls before and after matching for confounding variables.

To achieve this, we created two cohorts: a confounder-unmatched cohort and a matched cohort, based on whether the confounding variables matched. We reselected PDAC patients in a pairwise manner by identifying a control participant who was matched for values of each host metadata variables with only five of the 24 variables differed at most. In the same way, for ‘unmatched’ cohorts, patients and controls were unmatched for confounding variables as much as possible. Eventually, the matched and unmatched cohorts were composed of 50 samples with 25 cases and 25 controls respectively. Specific cohorts were shown in Table S4. We then conducted a series of statistical analyses to explore whether matching cases and controls for confounding variables could reduce observed differences in the microbiota. Our analysis of beta diversity revealed a significant difference in microbiota between PDAC patients and controls in the unmatched cohort (PERMANOVA, p = 0.001), but not in the matched cohort (p = 0.249) (Figure 1A). Similarly, the alpha diversity difference in unmatched cohort (Wilcoxon test, p = 0.002) was more significant than matched cohort (p = 0.099) (Figure 1B). Besides, we used Wilcoxon to test enriched taxa between PDAC and control (Figures 1C and 1D) and there were 5 taxa with p value below 0.001, 31 taxa with p value below 0.01, 88 taxa with p value below 0.05 in unmatched cohort, while 12 taxa with p value below 0.01, 83 taxa with p value below 0.05 in matched cohort (Figure S2). Additionally, we calculated the area under the receiver operating characteristic curve (AUROC) values by a 25-repeat stratified 4-fold cross-validation random forest for PDAC and controls before and after matching variables. According to the values of AUROC, the matched and unmatched cohort differed markedly by machine learning (Figure 1E). Overall, our results indicated that exposome played a significant role in shaping the composition of the gut microbiome in PDAC patients.

**Figure 2:**
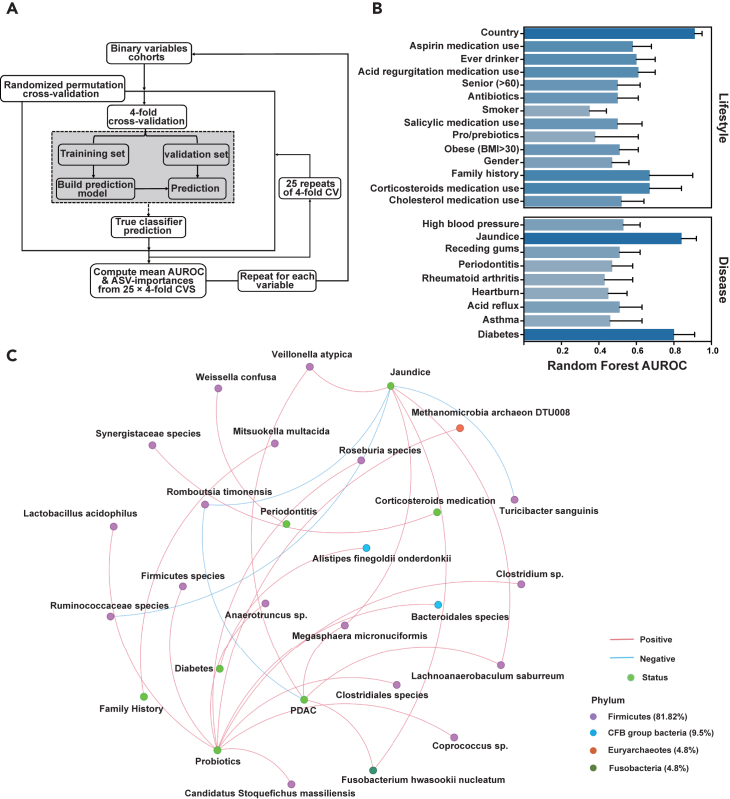
The random forest analysis framework and the significant association between species and exposome (A) The random forest analysis framework. The 25-repeat stratified 4-fold cross-validation over 75/25 splits was used for each binary variable. (B) The results of receiver operating characteristic (AUROC) for 23 binary lifestyle and disease variables. (C) Correlation network diagram showing the significant association between 21 species and 6 exposures. It was calculated using the Spearman correlation coefficient. The FDR was calculated using the Benjamin-Hochberg correction. Red line denotes a positive relationship, while blue line denotes a negative relationship.

We used the aforementioned random forest framework (Figure 2A) to predict binary variables by microbiome data in turn. The resulting values of AUROC (Figure 2B) revealed significant associations between microbiota and certain host variables, including jaundice, alcohol, acid regurgitation medication use, family history of PDAC, corticosteroids medication use, diabetes, and country (mean AUROC >0.6). Besides, we observed 21 significant associations (FDR_Spearman_ < 0.05) between 21 species and 6 exposures (Figure 2C and Table S1). For instance, the consumption of probiotics showed a significant positive correlation with the abundance of *Lactobacillus* species and *Clostridium* species. The cellular components and metabolites of these species play a crucial role in probiotic functions, primarily by activating gut epithelial cells and improving the integrity of the intestinal barrier.^36^

### Exposome‒microbiome mediation effects in PDAC

**Figure 3:**
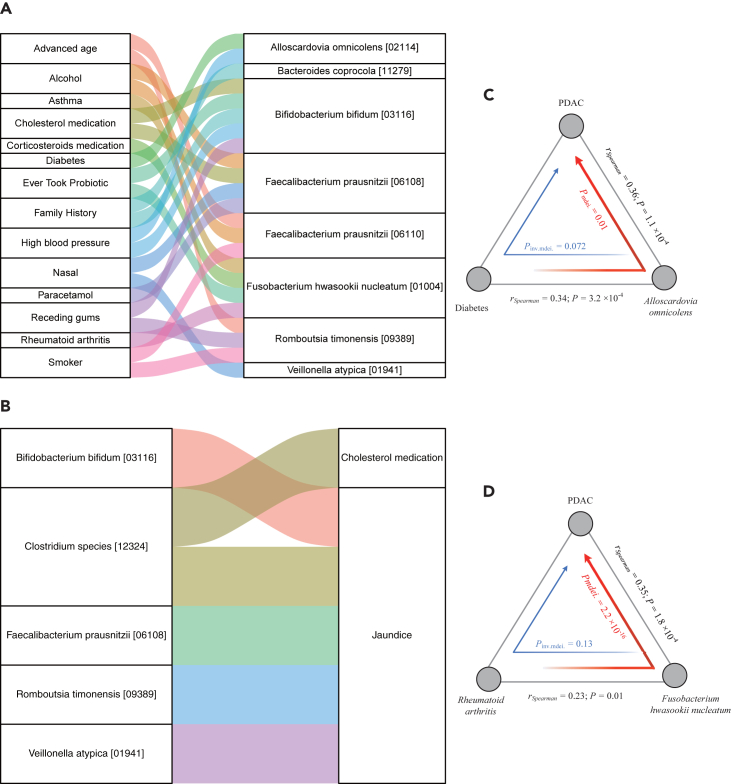
Mediation analysis identifies linkages between the microbiome, exposome and pancreatic cancer (A) Parallel coordinates chart showing the 23 mediation effects of exposome on PDAC through the microbiome, with significant level (FDR < 0.05). Shown are exposome (left), microbiome (right). The curved lines connecting the panels indicate the mediation effects. (B) Parallel coordinates chart showing the 6 mediation effects of the microbiome on PDAC through the exposome, with significant level (FDR < 0.05). Shown are microbiome (left), exposome (right). (C) Analysis of the effect of diabetes on PDAC as mediated by the abundance of Alloscardovia omnicolens. (D) Analysis of the effect of rheumatoid arthritis on PDAC as mediated by the abundance of Fusobacterium hwasookii nucleatum.

To explore the connections between exposome, the gut microbiome, and PDAC, we conducted a bi-directional mediation analysis using 23 exposures and 9 species that exhibited significant associations with PDAC (FDR_Spearman_ < 0.05). We identified a total of 29 mediating linkages (FDR_mediation_ < 0.05 & FDR_inverse-mediation_ > 0.05), with 23 involving the exposome impacting on PDAC through the microbiome, and 6 involving the microbiome impacting on PDAC through the exposome (Figures 3A and 3B). Most of these linkages were related to the impact of lifestyle factors and host disease on PDAC through microbiome. For example, we observed that diabetes can mediate the abundance of *Alloscardovia omnicolens*, thereby affecting the risk of PDAC (Figure 3C).

Rheumatoid arthritis (RA) is a chronic and systemic disease primarily characterized by inflammatory synovitis, the underlying cause of which remains unknown.^37^ *Fusobacterium hwasookii nucleatum* has the ability to activate the immune system and trigger inflammatory responses.^38^ In patients with RA, immune dysregulation and the progression of joint inflammation can potentially influence the composition of the microbial community, thereby contributing to an increase in *Fusobacterium hwasookii nucleatum* abundance. We observed that *Fusobacterium hwasookii nucleatum* can mediate the impact of rheumatoid arthritis on PDAC (Figure 3D; *P*_mediation_ = 2.2 × 10^−16^). Previously, Motasem et al. conducted a comprehensive nationwide study, which proposed that RA can manifest with extra-articular involvement in multiple organs, including the pancreas. Their findings revealed an elevated risk of pancreatic cancer among patients with RA, and those with a history of RA often exhibited a poorer prognosis.^39^

### The exposome is used in concert with gut microbiome data to predict pancreatic cancer

**Figure 4:**
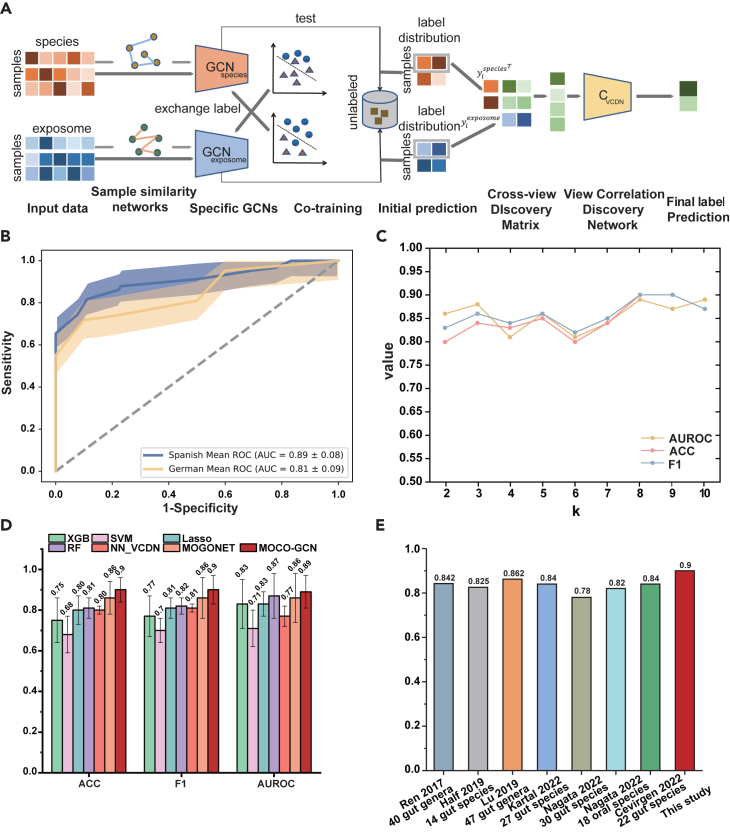
Illustration and performance of MOCO-GCN (A) The framework of MOCO-GCN. It combines of a Two-view Co-training Graph Convolutional Networks (GCNs) module that learns different omics data features by distilling knowledge from each other and a View Correlation Discovery Network (VCDN) module that integrates multi-omics data. Each species-GCN and exposome-GCN are trained to perform class prediction and the corresponding sample similarity network generated from the exposome and microbiome data. The co-training allows them to distill knowledge from each other by adding their most confident unlabeled data into the training set. The cross-omics discovery tensor is calculated from the initial predictions of omics-specific GCNs and forwarded to VCDN for final prediction. MOCO-GCN is an end-to-end model and all networks are trained jointly. (B) The performance of the MOCO-GCN on Spanish and German cohorts are shown as receiver operating characteristic (ROC) curve with 95% CI shaded in corresponding color. (C) Performance of MOCO-GCN under different values of hyper parameter k. (D) The comparison between MOCO-GCN and several traditional machine learning methods, including Support vector machine classifier (SVM), Linear regression trained with L2 regularization (Lasso), Random Forest classifier (RF), Gradient tree boosting-based classifier (XGBoost), and other multi-omics classification methods: MOGONET (Multi-Omics Graph Convolutional NETworks); NN_VCDN (fully connected NN with the same layers as the GCN in MOGONET). Data are represented as mean ± SEM. (E) The comparison between this study and previous studies that predict PDAC using gut microbiome alone.

To investigate the potential of combining exposome and gut microbiome data in predicting pancreatic cancer, we employed a framework called MOCO-GCN, which integrates predictions from both sources by leveraging their potential influence on the disease course and outcomes. MOCO-GCN is composed of a two-view co-training Graph Convolutional Networks (GCNs) and a View Correlation Discovery Network (VCDN) to classify PDAC and controls. The framework of MOCO-GCN is shown in Figure 4A. Specifically, co-training GCNs mainly predict initial labels with exposome and microbiome data by distilling knowledge from each other, while VCDN can effectively integrate initial labels by exploring the latent associations in the higher-level label space across exposome and microbiome data.^40^ To evaluate the performance of MOCO-GCN, we performed a 4-fold cross-validation, and assessed the final model performance using the average accuracy (ACC), average F1-score (F1), average AUROC, average Sensitivity (Sn), average Specificity (Sp), and average Matthews Correlation Coefficient (MCC).

We trained our model based on 23 exposures and 125 species selected through difference abundance analyses (Wilcoxon, p < 0.05; Table S2) and achieved excellent performance with 0.9 ± 0.06 ACC, 0.9± 0.07 F1, 0.89± 0.08 AUROC (Figure 4B), 0.86 ± 0.13 Sn, 0.93± 0.10 Sp, and 0.80± 0.11 MCC. Additionally, we conducted a sensitivity analysis that focused on the parameter k, which represents the average number of edges retained per node. Figure 4C illustrates the performance of MOCO-GCN as k varies from 2 to 10, demonstrating the stability of our model. We compared the performance of our model with several traditional machine learning methods, including Support vector machine classifier (SVM), Linear regression trained with L2 regularization (Lasso), Random Forest classifier (RF), Gradient tree boosting-based classifier (XGBoost), and other multi-omics classification methods: MOGONET (Multi-Omics Graph Convolutional NETworks);^41^ NN_VCDN (fully connected NN with the same layers as the GCN in MOGONET). These traditional machine learning methods were trained with the direct concatenation of the 125 species and 23 exposures as input. According to the classification results (Figure 4D), our model outperformed the previous methods and was more capable to predict pancreatic cancer with the integration of exposome and microbiome data.

According to the calculation of feature importance by our model, the top 45 features consist of three exposures, and 42 species are shown as a heatmap in Figure S3. Seventeen bacterial species were increased in the PDAC patients (n = 57) in comparison to those of the controls (n = 50), whereas 25 bacterial species were decreased. Among the 42 significantly important species, 26 (61.9%) belonged to the Firmicutes phylum, 7 (16.67%) belonged to Fusobacteria phylum, 3 (7.1%) belonged to CFB group bacteria phylum, 1 (2.4%) belonged to Actinobacteria phylum, 1 (2.4%) belonged to Basidiomycete fungi phylum, 3 (7.1%) belonged to High G + C Gram-positive bacteria class, and 1(2.4%) belonged to B-proteobacteria class. These results demonstrated the crucial role of the Firmicutes phylum in shaping the division between PDAC and controls. Species increased in the gut microbiomes of PDAC included *Fusobacterium hwasookii nucleatum*, *Alloscardovia omnicolens*, *Veillonella* spp. (*Veillonella atypica* and *Veillonella parvula*) and several unknown species in the phylum Firmicutes, while species depleted included several from the order *Clostridiales*, *Bacteroides coprocola*, *Faecalibacterium prausnitzii*, *Bifidobacterium bifidum*, and unknown Bacteroidales. Of note, our results were consistent with previous studies^16^^,^^17^^,^^18^^,^^19^^,^^42^ for 27 out of the 42 species investigated.

### Validation on an external cohort and comparison with previous studies

To evaluate the specificity of the trained models for PDAC, we assessed the accuracy of predictions using a dataset from a German study.^17^ This dataset consisted of 44 PDAC patients and 32 controls, with detailed information on 14 exposures. On the validation population from Germany, the MOCO-GCN model demonstrated a performance of 0.89 ± 0.07 in terms of accuracy (ACC), 0.91 ± 0.04 in terms of F1 score, and 0.81 ± 0.19 in terms of area under the receiver operating characteristic curve (AUROC) (Figure 4B). To further validate the performance of our model, we collected studies conducted within the past five years^16^^,^^17^^,^^18^^,^^19^^,^^43^ that investigated microbial prediction of pancreatic cancer. These studies primarily utilized traditional machine learning methods such as random forest and lasso regression. As illustrated in Figure 4E, our model exhibited superior predictive capabilities compared to previous studies by incorporating exposome data. These results collectively demonstrate the practicality and efficacy of MOCO-GCN in predicting pancreatic cancer by leveraging exposome and microbiome data.

## Discussion

This study represents an advancement in our understanding of the complex relationship between PDAC, microbiome, and exposome. Our findings provide compelling evidence for the influential role of exposome in microbiome-related studies of pancreatic cancer. We not only accurately predicted PDAC with the combination of microbiome and exposome, but also yielded important insights into the species and exposure level associations between these factors. First, our model demonstrated superior performance compared to other methods and previous studies, achieving satisfactory results on an external cohort. This underscores the importance of comprehensively considering the microbiome and exposome in PDAC-related research. Second, we emphasize the pivotal role of exposome in the interplay between PDAC and microbiome, highlighting the need to account for the specificity and correlation between these factors in future studies. Third, we assert that pancreatic cancer should not be regarded only as a localized disease, but rather as a systemic, environmental, and microenvironmental disease. Taken together, this study represents an important contribution to our understanding of the factors that contribute to PDAC development and underscores the importance of incorporating microbiome and exposome data in future research and clinical practice.

### Limitations of the study

This study provided valuable insights into the role of the microbiome and exposome in PDAC. However, it has several limitations that warrant acknowledgment. First, the small sample size and the challenging nature of collecting comprehensive gut microbiome and exposome data from a large population of pancreatic cancer patients highlight the need for more extensive longitudinal data to further elucidate the clinical translational and practical applications of this research. Second, the meta-variables data were limited to binary values, which restricted our ability to perform a more detailed analysis of factors such as alcohol consumption and smoking. Moreover, due to the lack of available data, we were unable to analyze two crucial risk factors for PDAC, exercise and diet. Third, despite the exceptional performance of our predictive model, the inherent complexity of deep learning models limits their interpretability, posing challenges in understanding the factors driving predictions. Therefore, more comprehensive follow-up research and analysis are required to clarify the mechanisms underlying PDAC as a microenvironmental and systemic disease. Addressing these limitations can provide a more comprehensive understanding of the factors contributing to PDAC development, informing the development of more effective prevention and treatment strategies. While our study primarily focuses on clinical aspects, we recognize the need for additional research to address challenges related to clinical costs and to advance practical applications in real-world medicine. In conclusion, our research provides initial methodologies and evidence for PDAC diagnosis based on microbiome and exposome data, but further research based on large-scale and more in-depth pancreatic cancer data are essential.

## STAR★Methods

### Key resources table

**Table:** 

REAGENT or RESOURCE	SOURCE	IDENTIFIER
**Deposited data**		

Raw sequencing data	Kartal et al.^17^	European Nucleotide Archive (ENA). Dataset identifiers: PRJEB38625; PRJEB42013

**Software and algorithms**		

Python (version 3.7.15)	Python Software Foundation	https://www.python.org/
Pandas (version 1.2.4)	Python package	RRID: SCR_018214; https://pandas.pydata.org/
PyTorch (version 1.11.0)	Python package	RRID: SCR_018536; https://pytorch.org/
scikit-learn (version 1.0.2)	Python package	RRID: SCR_002577; http://scikit-learn.org/
R (version 4.2.1)	R software	http://www.R-project.org
MOGONET	Wang et al.^41^	https://github.com/txWang/MOGONET
MOCO-GCN	This paper	https://github.com/Yuli-SDU/MOCO-GCN

### Resource availability

#### Lead contact

Further information and requests for resources should be directed to and will be fulfilled by the lead contact, Bingqiang Liu (e-mail: bingqiang@sdu.edu.cn).

#### Materials availability

This study did not generate new unique reagents.

#### Data and code availability

• All the raw data enrolled in this study have been deposited to the European Nucleotide Archive (ENA). The accession number is listed in the key resources table.
• All source code was available at https://github.com/Yuli-SDU/MOCO-GCN.
• Any additional information required to reanalyze the data reported in this paper is available from the lead contact upon request.

### Method details

#### MOCO-GCN

Microbiome and exposome integration strategies are needed to combine the complementary knowledge brought by each omics layer. The early integration used traditional machine learning methods based on the concatenation of every dataset into a single large matrix.^44^^,^^45^^,^^46^ This ignores the specific data distribution of each omic, which can potentially misguide ML models into finding irrelevant patterns that simply reflect the features’ membership to the same omics. To utilize the correlations across different classes and different omics data types to further boost the performance, we introduced MOCO-GCN, a framework for classification tasks with multi-omics data. The model is mainly composed of a Two-view Co-training Graph Convolutional Networks (GCNs) module for learning microbiome and exposome data features and improving the generalization ability of the GCN through the cooperation among multiple learners, and a View Correlation Discovery Network (VCDN)^40^ module for multi-omics data integration. The detailed architecture of the model for predicting pancreatic cancer in this study is shown in Figure 4A. First, for exposome and microbiome data, we constructed ${GCN}_{species}$ and ${GCN}_{exposome}$ based on weighted sample similarity networks using cosine similarity, to train specific omics data. Then, we used co-training to train GCN on two different views and exchange labels of unlabeled instances in an iterative way. Consequently, the initial predictions of class labels were generated by the co-training of ${GCN}_{species}$ and ${GCN}_{exposome}$. Secondly, we constructed the cross-omics discovery tensor which integrated the ${GCN}_{species}$ and ${GCN}_{exposome}$ label correlations. And after reshaping into a vector, it was forwarded to VCDN for final label prediction. MOCO-GCN is an end-to-end model, and every module is trained alternatively until convergence.

#### Two-view Co-training GCNs

First, we used cosine similarity for exposome and microbiome data respectively to construct sample similarity networks. The cosine similarity calculation formula is as follows:

(Equation 1) $$similarity=\cos\left(\theta \right)=\frac{A\cdot B}{\left\|A\right\|\left\|B\right\|}=\frac{\sum_{i=1}^{n}A_{i}\times B_{i}}{\sqrt{\sum_{i=1}^{n}{\left(A_{i}\right)}^{2}}\times \sqrt{\sum_{i=1}^{n}{\left(B_{i}\right)}^{2}}}$$

Where A and B are the two attribute vectors of the sample about the microbiome and the exposome. And each participant is a node in the sample similarity network.

Further, we constructed the feature matrix and the graph structure as the inputs in GCN model. Of these, the feature matrix is defined as $X\;\epsilon {\;R}^{n\times d}$, where n is the number of nodes and d is the number of features. And the graph structure is expressed in the form of adjacency matrix $A\;\epsilon {\;R}^{n\times n}$, where A is constructed by calculating the cosine similarity between nodes:

(Equation 2) $$A_{ij}=\left\{ \begin{array}{c} s\left(x_{i},x_{j}\right),if\;i\neq j\;and\;s\left(x_{i},x_{j}\right)\geq \varepsilon \\ 0,otherwise \end{array}\right.$$

Where $A_{ij}$ represents the adjacency between node i and node j, $x_{i}$ and $x_{j}$ are the feature vectors of node i and node j. ε is the threshold determined by k, which is the average number of edges retained per node:

(Equation 3) $$k=\sum_{i,j}I\left(s\left(x_{i},x_{j}\right)\geq \varepsilon \right)/n$$

Where I(·) is the indicator function and note that for the parameter k=1, A would contain no edge and a GCN would degenerate to a normal neural network (NN). A proper k value is important for the performance of MOCO-GCN. Figure 4C shows the performance of MOCO-GCN when k varies from 2 to 10.

Finally, we built GCN by stacking multiple convolutional layers, each layer is defined as:

(Equation 4) $$H^{\left(l+1\right)}=f\left(H^{\left(l\right)},A\right)=\sigma \left({\hat{D}}^{-\frac{1}{2}}\left(A+I\right){\hat{D}}^{-\frac{1}{2}}H^{\left(l\right)}W^{\left(l\right)}\right)$$

Where l is the number of layers in the convolutional layer of the graph, $H^{\left(l\right)}$ is the input of layer lth, $W^{\left(l\right)}$ is the weight matrix of layer lth, $H^{\left(l+1\right)}$ is the output of layer lth, $\hat{D}$ is the diagonal node degree matrix of A and I is the identity matrix. σ(·) represents a non-linear activation function.

To overcome the limits of the GCN model with shallow architectures, we introduce co-training and self-training to train GCNs. As a method of multi-view learning, co-training explores the effective information in unlabeled data and improves the generalization ability of the model through cooperation among multiple learners.^47^ Given the labeled data $y_{i}\left(i=1,2,3,...\right)$ and the unlabeled data $y_{j}\left(j=1,2,3,...\right)$, the predictions $y_{i}^{species}$, $y_{i}^{exposome}$ under two different views could be obtained by different learners ${GCN}_{species}$ and ${GCN}_{exposome}$. The classifiers are used to estimate the label confidence of the unlabeled samples, and the trusted samples are added to the training dataset for iterative training to optimize the classifiers. This allows them to distill knowledge from each other by adding their most confident unlabeled data into the training set. Once all the unlabeled samples are labeled with each trained model and ${GCN}_{species}$ and ${GCN}_{exposome}$ become stable, the training ends.

#### VCDN for label integration

VCDN is proposed to fully explore the cross-view label correlations and improve model performance. After the initial classification results $y_{i}^{species}\epsilon \;R^{2}$ and $y_{i}^{exposome}\epsilon \;R^{2}$ are achieved by the co-training of ${GCN}_{species}$ and ${GCN}_{exposome}$, the cross-view label-level adjacency matrix $c_{i}\in R^{2\times 2}$ is defined as:

(Equation 5) $$c_{i}=y_{i}^{\text{exposome}}\cdot y_{i}^{\text{specie}s^{T}}$$

Then, $c_{i}$ is reshaped to a $2^{2}-dimensional$ vector and forwarded to $C_{VCDN}\left(\cdot \right)$ to predict the final prediction, notice that $C_{VCDN}\left(\cdot \right)$ is denoted as a fully connected network with an output dimension of 2.^40^

The loss function of MOCO-GCN is defined as:

(Equation 6) $$L=L_{GCN}^{\text{species}}+L_{GCN}^{\text{exposome}}+L_{VCDN}$$

Of these, $L_{GCN}$ could be written as:

(Equation 7) $$L_{GCN}^{j}=\sum_{i=1}^{n}L_{CE}\left({\hat{y}}_{i}^{j},y_{i}\right)=\sum_{i=1}^{n}-\log\frac{e^{{\hat{y}}_{i}^{j},y_{i}}}{\sum_{k}e^{{\hat{y}}_{i,k}^{j}}}$$

Where $y_{i}\;\epsilon \;R^{n}$ is the ground-truth label vector of ith sample and $L_{CE}\left(\cdot \right)$ is the cross-entropy loss function. ${\hat{y}}_{i}^{j}$ represents the predicted label probability of ith training sample, notice that j∈(species,exposome). And $L_{VCDN}$ could be written as:

(Equation 8) $$L_{VCDN}=\sum_{i=1}^{n}L_{CE}\left(VCDN\left(c_{i}\right),y_{i}\right)$$

We fix VCDN(·) and update ${GCN}_{i}\left(\cdot \right)$, i= 1, 2, 3, 4 for microbiome and exposome data to minimize the loss function L.

At last, to verify the performance of MOCO-GCN, we used the 4-fold cross-validation method to compare the model with the traditional machine learning models, such as Support vector machine classifier (SVM), Linear regression trained with L2 regularization (Lasso),^17^ Random Forest classifier (RF),^16^ Gradient tree boosting-based classifier (XGBoost), and other multi-omics classification methods: MOGONET (Multi-Omics Graph Convolutional NETworks);^41^ NN_VCDN (fully connected NN with the same layers as the GCN in MOGONET). The evaluation index consists of accuracy (ACC), F1score (F1), and AUROC. We reported the mean and standard deviation results of the 4-fold cross-validation. All above framework was implemented in Python 3.10 with numpy, pandas, matplotlib, scikit-learn package, KFold, XGBClassifier, LogisticRegression, RandomForestClassifier, SVC, and torch.

#### Identifying biomarkers with MOCO-GCN

To determine the most important microbiome and exposures in the process of diagnosing PDAC, we employed a feature ablation method^48^ to calculate feature importance. Using the change in F1 score as the metric, we assigned each feature to zero and calculated the decrease in MOCO-GCN performance on the test set compared to using all the features. Features that exhibited the greatest performance drop were considered to be the most important ones. In essence, the measurement involved comparing the difference in model performance after excluding each feature with the original performance.

### Quantification and statistical analysis

#### Alpha and beta diversity analysis

The Shannon diversity^49^ was used as a measure of within-individual diversity, and this was calculated using the R function “diversity”. The beta diversity^50^ was used as a measure of between-sample differences in community composition and was quantified as Bray-Curtis dissimilarity. The principal coordinate analysis (PCoA) was used to sort and visualize the beta diversity matrices. Differences in alpha diversity between groups were examined with Wilcoxon rank-sum test (Figure 2B). Differences in beta diversity between groups were tested using the ‘adonis2’ implementation of permutational multivariate analysis of variance (PERMANOVA) (Figure 2A). The R packages used for calculation and visualization for all analyses included: vegan, ggplot2, ade4, RcolorBrewer, dplyr, and reshape2.

#### Difference abundance analysis

The difference abundance analyses in PDAC cases and controls were implemented by Wilcoxon rank-sum test with the R function “wilcox.test”. And we used the generalized fold change (GFOLD) to figure out the microbial abundance fold change between PDAC cases and controls. The value of generalized fold change greater than 0 indicates enrichment in PDAC, while less than 0 indicates enrichment in controls.

#### Machine learning

To identify the microbiota-associated variable and find the microbiota community difference between PDAC patients and controls by machine-learning analyses, we constructed a random forest classifier of 25-repeat stratified fourfold cross-validation which leveraged bootstrapping and cross-validation to identify groups. The general pipeline is shown in Figure 3A. The training and testing occurred on separate, randomly selected, stratified sampling splits of 75% and 25% of the data. It was set to have 512 decision trees and one sample for each leaf so that the problem of overfitting on the training set would be eliminated which applied to few samples relative to the number of ASVs. The mean-AUROC value was from the average of 100 repeats. All above framework was implemented in Python 3.10 with numpy, pandas, matplotlib, and scikit-learn package.

#### Bi-directional mediation analysis

To revealed putative causal relationships between PDAC, the microbiome and exposome, we implemented a bi-directional mediation analysis. This analytical approach allowed us to investigate the mediating effects of the microbiome and exposome on the development and progression of PDAC. We focused on 23 exposures and 9 species that exhibited significant associations with PDAC (FDR_Spearman_ < 0.05). The FDR was calculated using the Benjamini–Hochberg procedure. we conducted a bi-directional mediation analysis with interactions, employing the equation y = x + m + x × m, where y represents the outcome, x represents the independent variable, and m represents the mediator. All of these analyses were performed using the mediation package in R.

## Supplemental information

Document S1. Figures S1–S3

Table S1. Spearman Rank Correlation Results, related to Figure 3

Table S2. The results of differential abundant test between PDAC and controls, related to Figure 1

Table S3. Detailed interpretation of exposome data, related to Figure 2

Table S4. The IDs of matched and unmatched cohort, related to Figure 1
