# Supplementary material for: Highly accurate diagnosis of pancreatic cancer by integrative modeling using gut microbiome and exposome data
Source: iScience. 2024 Feb 21;27(3):109294. doi: 10.1016/j.isci.2024.109294 (PMC10915599; doi:10.1016/j.isci.2024.109294)
Supplement: Document S1. Figures S1–S3 [file mmc1.pdf]

iScience, Volume *27*

Supplemental information

Highly accurate diagnosis of pancreatic cancer by integrative modeling using gut microbiome and exposome data

Yuli Zhang, Haohong Zhang, Bingqiang Liu, and Kang Ning

Supplemental information

Highly accurate diagnosis of pancreatic cancer by integrative modeling using gut microbiome and exposome data

Yuli Zhang, Haohong Zhang, Bingqiang Liu, Kang Ning

Supplemental information

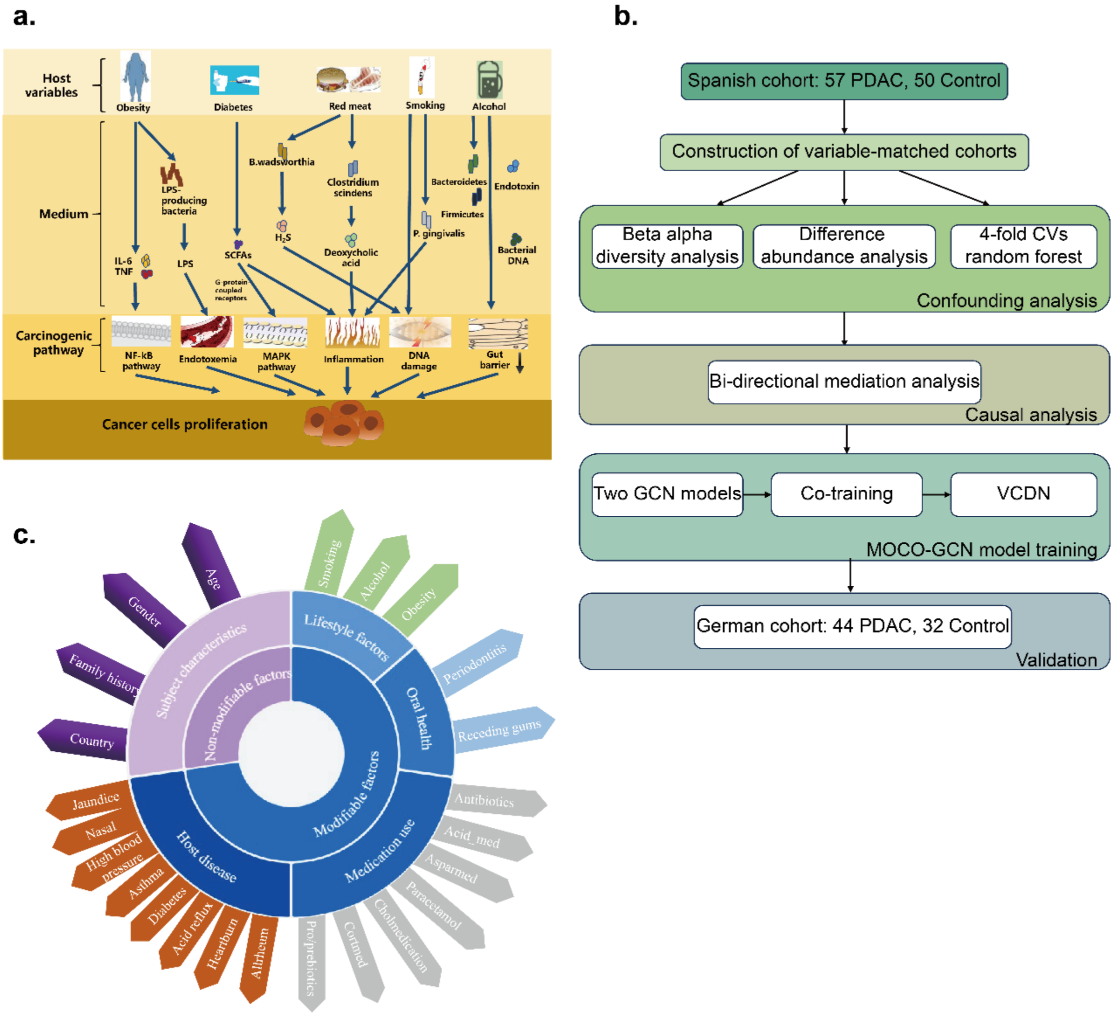

Figure S1. Pathways of exposures and microbiome interact to influence pancreatic carcinogenesis and the establishment of host metadata, related to Figure 2. (a) Pathways by which dietary, host disease, and lifestyles affect the gut microbiome and their role in PDAC carcinogenesis. For example, obesity and alcohol may promote PDAC through LPS-mediated systemic inflammation and dysbiosis of Bacteroidetes and Firmicutes. **(b)** Analysis workflow. CV: Cross-validation. GCN: Graph Convolutional Networks. MOCO-GCN: Multi-Omics Co-training Graph Convolutional Networks. VCDN: View Correlation Discovery Network. **(c)** The establishment of host variables in our data. It is mainly composed of non-modifiable factors which include age, gender, family history, and country, and modifiable factors which include host disease, medication use, oral health, and lifestyle factors.

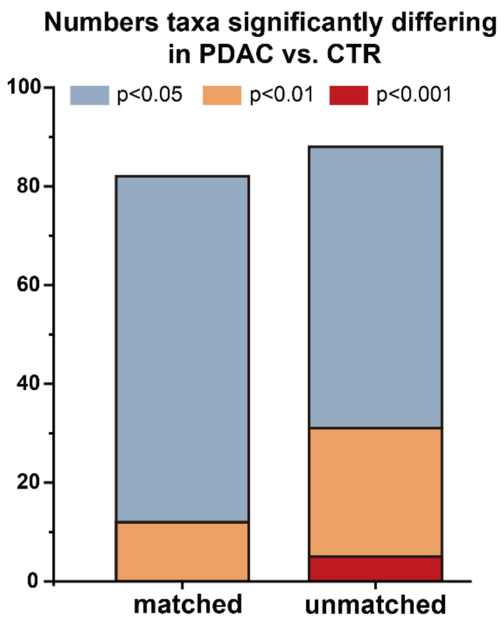

Figure S2. The numbers of taxa significantly differed from PDAC and controls in the confounding-matched and unmatched cohort, related to Figure 1.

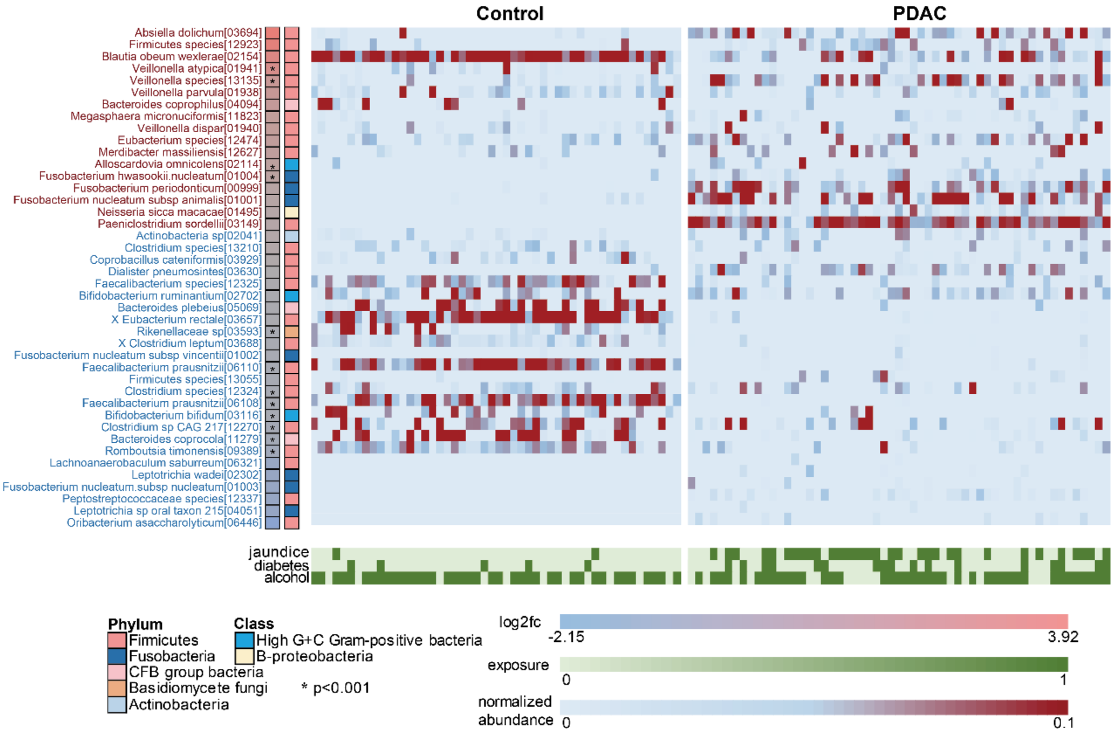

Figure S3. **Normalized abundance of top 42 selected species in the gut microbiome and 3 exposures across samples shown as a heat map, related to Figure 4**. A value of generalized fold change (log2fc) greater than zero indicates enrichment in PDAC, and the font color of species is red; otherwise, enrichment in controls, and the species font is blue.
